# The risk of imported malaria in security forces personnel returning from overseas missions in the context of prevention of re-introduction of malaria to Sri Lanka

**DOI:** 10.1186/s12936-016-1204-y

**Published:** 2016-03-08

**Authors:** Sumadhya Deepika Fernando, Priyani Dharmawardana, Saveen Semege, Geetha Epasinghe, Niroshana Senanayake, Chaturaka Rodrigo, Risintha Premaratne

**Affiliations:** Department of Parasitology, Faculty of Medicine, University of Colombo, 25, Kynsey Road, Colombo 08, Sri Lanka; Anti Malaria Campaign, 555/5 Public Health Building Complex, Narahenpita, Colombo 5, Sri Lanka; Sri Lanka Army Health Services, Army Headquarters, Baladaksha Mawatha, Colombo 03, Sri Lanka; Department of Clinical Medicine, Faculty of Medicine, University of Colombo, Colombo, Sri Lanka

**Keywords:** Malaria, Chemoprophylaxis, Malaria elimination, Prevention of re-introduction, Sri Lanka, Security forces

## Abstract

**Background:**

Sri Lanka is a malaria-free country. However it remains surrounded by countries with endemic malaria transmission. Since the last indigenous case of malaria was reported in October 2012, only imported malaria cases have been diagnosed with 36 cases detected in 2015, which includes 17 cases each of *Plasmodium vivax* and *Plasmodium falciparum* and two cases of *Plasmodium ovale*.

**Methods:**

This study investigated the knowledge and practices regarding malaria chemoprophylaxis among all the Sri Lankan security forces personnel returning from peacekeeping missions in malaria endemic countries over a 7 month period. Adherence to other malaria prevention measures, occurrence of adverse events and incident cases of malaria were also recorded maintaining the anonymity of the respondents. Potential associations for non-compliance were studied.

**Results:**

Interviews were carried out with 559 security forces personnel returning home from foreign deployments in malaria-endemic regions (males: 550, 98.4 %). The majority (553, 98.9 %) was well aware of the need for chemoprophylaxis during the overseas stay and its regular use as prescribed. The overall adherence to chemoprophylaxis was good with 78.7 % (440/559) reporting regular, as prescribed, use. Having better educational qualifications, being female, being prescribed mefloquine, having fever during deployment and belonging to a security force other than the army were significantly associated with poor compliance (p < 0.05).

**Conclusions:**

The study reveals that knowledge regarding malaria chemoprophylaxis among Sri Lankan security forces personnel serving abroad was good, a fact that may have contributed to absence/extremely low incidence of malaria during deployment.

## Background

Malaria has made a comeback in many of the areas from which it was previously eliminated during the Global Malaria Eradication Programme. This serves as a reminder that vigilant systems need to be sustained for as long as the mosquito vectors, a suitable climate and other conditions exist to facilitate disease transmission [1]. The risk of resurgence is determined by the prevailing vectorial capacity (receptivity), the malaria importation rate (vulnerability), and the malariogenic potential [2–4]. Therefore, malaria elimination, once achieved, is more likely to be sustained in regions where receptivity is low, or in geographically isolated areas with a lower risk of imported malaria [4–6].

Sri Lanka was gripped by a separatist war for nearly 30 years until 2009, which affected mainly the Northern and Eastern parts of the country, leading to frequent interruptions of healthcare services in these areas. Approximately 60 % (189, 283/336, 640) of malaria cases reported in Sri Lanka between the years 2000 and 2012 were from these areas. Although there has been a decline in malaria cases in the country during the last 15 years, the number reported among the security personnel remained disproportionately high. In the last 5 years prior to elimination of local transmission (2008–2012), 2059 indigenous malaria cases were reported in Sri Lanka and 1547 of these (75 %) were from the members of the security forces [7]. The conflict ended in 2009 and the government had uninterrupted access to these regions to continue its malaria elimination activities. With that the last indigenous case of malaria in Sri Lanka was reported in October 2012.

Sri Lanka commenced reporting imported malaria cases separately since 2008 and the highest number of cases (95) was reported in 2013. Malaria surveillance takes place to identify early and treat such cases. The majority of the cases being imported to Sri Lanka have so far originated in India, Pakistan and the continent of Africa [8]. Currently security forces personnel serve on United Nations peacekeeping missions in malaria endemic countries in Africa, South East Asia and the Caribbean (Haiti). They also attend training courses in India, Bangladesh and Pakistan which report a high number of malaria cases annually. This has led to a number of imported malaria cases being reported from the security forces [9]. With a higher proportion of security forces personnel involved in missions in malaria endemic countries, there has been a considerable risk of importation of malaria to Sri Lanka.

Malaria chemoprophylaxis is issued free-of-charge to travellers by the Anti Malaria Campaign (AMC) and remains the most important strategy for preventing malaria in security forces personnel. The medicines currently recommended for chemoprophylaxis by AMC are mefloquine 250 mg weekly for those visiting African countries and chloroquine base 300 mg weekly for Indian subcontinent destinations and Haiti [10].

The objectives of this study were to assess the knowledge and practices with respect to malaria prevention among Sri Lankan security forces personnel travelling to malaria endemic countries. Rates of compliance with chemoprophylaxis were also investigated in security forces personnel returning from their missions and rates of self-reported adverse events from chemoprophylaxis.

## Methods

This was a cross sectional study among security forces personnel who returned to the country after serving or training in malaria endemic countries. The security forces personnel included in this paper were members of Sri Lankan Army, Air Force, Police and Special Task Force (a special unit of the police that are directly deployed in combat zones) who were deployed in United Nations peacekeeping missions abroad. The calculated minimal sample size required was 405 after allowing for a 5 % non-response rate [11]. However, all returning security forces personnel within the period of study (March 2015–September 2015) were included in the study, with a view of further increasing the power of the study as well as allowing sub-group analysis. Returnees were interviewed within 2 weeks of their arrival to Sri Lanka.

Following the return of security forces personnel from a malaria endemic country, the coordinators of the armed forces arranged a meeting with the investigators. During this interview, data was collected using an interviewer-administered questionnaire after obtaining informed written consent from the participant. Awareness of chemoprophylaxis, self reported adherence to chemoprophylaxis, occurrence of adverse events (AEs), mosquito bite prevention methods utilized abroad and getting tested for malaria following return to Sri Lanka were evaluated using a questionnaire with multiple choices. For some questions if the answer was not available within the given choices, a space was left to write the appropriate answer/opinion. If an individual reported any neuropsychiatric adverse effects, medically qualified investigators were assigned to interview them further. The questionnaire was pre-tested on 25 security forces personnel who had returned to Sri Lanka prior to the commencement of the study.

Chemoprophylaxis was considered as regular if the medicines have been taken for the whole duration of overseas stay at the recommended dose and intervals; irregular, if chemoprophylaxis had been taken at a frequency less than recommended, and interrupted or not performed, if chemoprophylaxis had never begun, or had been started but subsequently interrupted.

Ethical approval for the study was obtained from the Ethics Review Committee of the Faculty of Medicine, University of Colombo. Permission for the study was also obtained from the Director General (Army Health Services), The Commanders of the Air Force and the Inspector General of Police. The privacy of respondents was ensured during the conduct of interviews. It was clearly explained to each of them that disclosing details was not a mandatory requirement and that information would not be disclosed to a third party including their commanding officers. The information from the interviews was not traceable to individual participants.

Data was analysed using SPSS (Version 20, IBM SPSS Statistics, USA) software package. Frequencies, proportions, means and standard deviations were used for the descriptive analysis. Significance of associations for poor compliance was assessed using Chi square test or Fishers exact test, with statistical significance set at 0.05. The independent variables were arranged as dichotomous categories (degree of freedom − 1) for comparison in Chi square test. The associations were also expressed as a risk ratio (± 95 % confidence intervals) using the same 2 × 2 table.

## Results

### Demography and nature of foreign deployment

The study population comprised 559 security forces personnel returning home after foreign deployments in malaria endemic overseas territories (males: 550, 98.4 %). There were no instances of refusal of consent. The majority (538/559, 96.2 %) was from the Sri Lanka Army. The most common foreign station of deployment was Haiti (489/559, 87.5 %) and the duration of stay was less than 6 months for many (546/559, 97.7 %). Other details on demography and overseas deployment are summarized in Table 1.

**Table 1:** Demography and details of foreign deployment of the sample (n–559)

Characteristic	Number (%)
Sex	
Male	550 (98.4)
Female	9 (1.6)
Ethnicity	
Sinhala	559 (100)
Highest educational qualification	
Up to grade 10	250 (44.7)
Passed G.C.E. O/L examination^a^	207 (37)
Passed G.C.E. A/L examination^a^	60 (10.7)
Diploma	21 (3.8)
Graduate	16 (2.9)
Postgraduate	5 (0.9)
Armed force	
Army	538 (96.2)
Other forces	21 (3.8)
Rank	
Other ranks	439 (78.5)
Officers	120 (21.5)
Duration of service in the armed services (years)	
<5 years	3 (0.5)
6–10 years	224 (40.1)
11–15 years	86 (15.4)
16–20 years	166 (29.7)
21–25 years	66 (11.8)
>25 years	14 (2.5)
Foreign country of most recent deployment	
Haiti	489 (87.5)
South Sudan	63 (11.3)
Liberia	6 (1.1)
Democratic republic of congo	1 (0.2)
Duration of stay on foreign mission	
>6 months	546 (97.7)
6–12 months	12 (2.1)
18–24 months	1 (0.2)

^a^G.C.E O/L and A/L—General Certificate of Education Ordinary and Advance level examinations, held at 11th and 13th grades of school respectively. The latter is the final examination of school education

### Risk factors for malaria

Of the entire sample, two gave a history of confirmed malaria during their overseas stay (2/559, 0.4 %), one of them acquiring the disease twice. Over 90 % had come into contact with a person with malaria within the preceding 3 months (520/559). None gave a history of a blood transfusion within the preceding 3 months.

### Knowledge and practices regarding malaria chemoprophylaxis

A majority (553/559, 98.9 %) of the sample were well aware of the need to start chemoprophylaxis prior to departure and the need for its regular use as prescribed during the overseas stay. However, more than 80 % (459/559) were unaware that the medication had to be continued for a specified period of time following return to Sri Lanka. Chloroquine had been dispensed to 98.5 % of security forces personnel belonging to the Army and Air Force and mefloquine was given to eight of twelve individuals serving in other forces during their period of service in a foreign country. The overall adherence to chemoprophylaxis was good with 78.7 % (440/559) reporting regular use as prescribed. Of the 110 security forces personnel who did not take the medicine regularly as instructed, forgetfulness was the main reason for intermittent use (77/110, 70 %). Of the nine individuals who did not take prophylaxis at all, five did so despite having access to prophylaxis and four did not receive prophylaxis prior to departure or at their overseas destination. Few individuals with poor compliance (any pattern of use other than regular use) (24/115, 20.9 %), reported non-use due to fear of adverse effects. In spite of a widely held perception that malaria chemoprophylaxis is not being taken due to the fear of impotency, only five individuals gave this as a reason for not taking the drug regularly or not at all.

Of other preventive practices during deployment, the most popular methods were the use of mosquito nets, mosquito repellents and long sleeved clothing. A majority (522/559, 93.4 %) had used one or more of these alternative measures to avoid mosquito bites. Only 37 (37/559, 6.6 %) said that they did not use any measures to avoid mosquito bites. The majority was aware that upon return, their blood had to be tested for malaria parasites (487/559, 87.1 %) and 550 (550/559, 98.4 %) had their blood tested upon return on the advice of the authorities. Since the coordinators of the armed forces inform the Anti Malaria Campaign officials of the arrival of troops from overseas, 99.2 % (546/550) of these tests had been conducted at the airport itself on arrival. All nine individuals that missed testing were from one particular force and none of them were aware of the need to do a blood test for malaria on their return to Sri Lanka. Other details regarding knowledge and practices on malaria chemoprophylaxis are summarized in Table 2.

**Table 2:** Knowledge and practices regarding malaria chemoprophylaxis and surveillance in the sample (n–559)

Knowledge and practices	Number (%)
*Compliance with chemoprophylaxis*	
Regular (as prescribed)	440 (78.7)
Intermittent	110 (19.7)
Not taken	5 (0.9)
Medicines were not provided	4 (0.7)
*Source of medicines*	
Doctors of the armed forces	502 (90.4)
Anti Malaria Campaign	7 (1.3)
Doctors of United Nations	5 (0.9)
Nursing officers in the field	8 (1.4)
Pharmacists in the field	6 (1.1)
Other	27 (4.8)
*Usage pattern of chemoprophylaxis*	
First dose taken before departure	520 (94.5)
Change of chemoprophylaxis	6 (1.1)
Continuation of medication following return	31 (5.5)
*Usage of other methods to avoid malaria*	
None	37 (6.6)
Mosquito nets	492 (94.2)
Mosquito repellents and coils	193 (36.9)
Long sleeved cloths	120 (22.9)
Destroy breeding places of mosquitoes	266 (50.9)

### Adverse effects

Only 12 (12/550, 2.1 %) of the participants reported adverse effects attributable to taking chemoprophylaxis regularly. They were all minor reactions including nausea (2/12, 16.6 %) and headache (5, 41.6 %), which did not restrict them from continuing to take the medicine. No neuropsychiatric adverse effects were reported from the eight personnel who took mefloquine.

### Malaria infections

Twenty-three (23/559, 4.1 %) reported having had fever during their deployment abroad. Two had been diagnosed with malaria and both of them were deployed in Liberia for 12 months. One of the above had contracted *Plasmodium falciparum* malaria twice during deployment. The type of malaria was unknown in the second person. Of these two individuals one had poor knowledge of chemoprophylaxis and had not taken regular chemoprophylaxis during or after the deployment. The other had no knowledge of chemoprophylaxis and had not taken medicines at all to prevent malaria. Neither had been tested for malaria following their return.

### Associations of poor compliance with chemoprophylaxis

Being female, having a better education (having passed at least G.C.E. O/L examination), being on mefloquine, having fever during deployment, belonging to a security force other than the Army were significantly associated with poor compliance (p < 0.05). Poor compliance was defined as not having regular use of chemoprophylaxis as prescribed. Duration of service, rank in the military, source of prescription and starting the first dose prior to departure were not significantly associated with compliance (Table 3).

**Table 3:** Associations for poor compliance (any pattern of use other than regular use as prescribed)

Comparison	Risk ratio (95 % confidence interval), p value*
Being male	0.37 (0.2–0.68), 0.02, s
Having passed at least the G.C.E. O/L examination** (yes vs. no)	1.6 (1.13–2.25), 0.01, s
Officers vs. others	1.08 (0.96–1.21), 0.207, ns
Army vs. others	0.32 (0.23–0.46), <0.001, s
Duration of service 10 years or less vs. others	1.03 (0.95–1.13), 0.528, ns
Being on chloroquine (yes vs. no)	0.27 (0.19–0.39), <0.001, s
Prescribed by an armed forces doctor vs. others	1.02 (0.88–1.18), 0.86, ns
Receiving first dose before departure (yes vs. no)	1.19 (0.96–1.5), 0.068, ns
Having fever during deployment (yes vs. no)	1.9 (1.12–3.26), 0.04, s

*ns* not significant, *s* significant

** General Certificate of Education—ordinary level

## Discussion

The strengths and weaknesses of available strategies to prevent imported malaria through security forces personnel returning to Sri Lanka after foreign missions are discussed in this manuscript. There had been no cases of indigenously acquired malaria in Sri Lanka for over 3 years. Elimination of malaria is not a one-off achievement, and constant effort and vigilance is necessary to maintain the malaria-free status. The lessons learnt from the island nation’s experience in both elimination as well as post-elimination surveillance could be valuable for other countries in their elimination efforts.

In this regard, this study has been monitoring the transition of Sri Lanka to a malaria-free nation in collaboration with the National Anti-Malaria campaign and have previously published on key strategies to prevent the re-introduction by educating population sub groups at a high risk of acquiring malaria as well as key response groups. These included security forces personnel serving in previously endemic areas of the country and medical officers serving in previously endemic regions [12, 13].

Security forces are considered a high-risk group for imported malaria as large contingencies of Sri Lankan security forces personnel have been serving on a rotational basis in United Nations peace-keeping missions in malaria endemic countries. The socio-economic and political turmoils at these locations leave them at increased risk of being exposed to malaria [14]. Resistance to first-line anti-malarials are prevalent in many African countries (e.g. Central African Republic) the troops are deployed [15]. This increases the risk of treatment failure and re-introduction of malaria into Sri Lanka.

This study shows that at present, measures to prevent importation of malaria by security forces personnel travelling abroad are very satisfactory in most aspects. These include; raising awareness of the need for prophylaxis, ensuring compliance with chemoprophylaxis and screening upon re-entry into the country. However, some deficiencies were also noted which can be rectified easily by adding extra surveillance steps and health education to the existing infrastructure (e.g. educating on the need to continue prophylaxis following return to the country and continued follow up for malaria for 3–6 months after return).

Being in the army was significantly associated with better compliance with chemoprophylaxis in this study compared to other security forces. None of the Sri Lankan Army personnel had contracted malaria during their overseas deployment. The two persons who got infected and all individuals who had not been tested upon return to the country belonged to another security force group which suggests the need of a better mechanism to educate all security forces personnel serving in Sri Lanka on malaria. In the year ending on October 2015, the Sri Lanka Army had sent 1058 of its servicemen and women to missions abroad. A majority (916/1058, 86.6 %) of them had been covered by the Army’s medical units on malaria prophylaxis. The administration of prophylaxis is done every Sunday under a direct supervision of a medical officer. Despite the large number of overseas deployments, the army has reported only five cases of malaria for the last 5 years among its troops in overseas deployment (Personnel Communication, Public Health Specialist, Sri Lanka Army). The AMC is notified 2 weeks prior to the return of soldiers, due to the close liaison which exists between the two organizations. Screening for malaria is carried out by both AMC medical officers and the Army medical corps at the airport.

Being female and mefloquine use were both associated with poor compliance in this study. However, the numbers in both these categories were few. It is difficult to assume the reasons for this observation though it is plausible that fear of adverse effects with mefloquine (as opposed to the time-tested and popular chloroquine) may be a reason. Mefloquine administration for chemoprophylaxis has recently come under criticism after significant proportion of Australian Defence Force personnel deployed in East Timor complained of neuropsychiatric adverse events after taking the drug [16, 17]. Members of Sri Lankan Army deployed to South Sudan, received chloroquine (It has less side effects but is not a recommended prophylactic agent for this location [18]) due to mefloquine unavailability.

It is interesting that having a better education was inversely associated with better compliance in this study. Here, it is possible that those with a lower level of educational qualifications belong to lower ranks, which are under strict supervision to take prophylaxis. There was no association with belonging to an officer’s rank and compliance but the study only examined up to the level of a Lieutenant.

Several interesting observations emerge from similar studies on malaria chemoprophylaxis in other countries. A large scale cross sectional study of peacekeeping forces deployed in Afghanistan from 2002 to 2011 evaluated the compliance with mefloquine in 5773 participants [19]. The compliance was approximately 80 % until 2006 and dropped to less than 60 % between 2007 and 2011. Adverse events were reported by 21.2 % (875/5773) of recruits but none were serious. Regular prophylaxis was interrupted only in 113 (113/5773, 2.7 %) subjects due to adverse effects. No malaria cases were reported during deployment in this study. The authors concluded that mefloquine was safe for large-scale administration and was efficacious even when the overall uptake was not optimal. Another study on chemoprophylaxis for soldiers in Kenya compared doxycycline against mefloquine on the interruption of capacity to work due to adverse effects [20]. The doxycycline group complained significantly more about interruptions to work due to adverse effects. However, this study was based on self-reported adverse effect profiles. A study on US troops stationed in Afghanistan between 2006 and 2007 interviewed 2351 respondents and the compliance with mefloquine was better than that with doxycycline (80 vs. 60 %). A larger proportion discontinued doxycycline compared to mefloquine (10 vs. 4 %). Doxycycline was unpopular due to its dosing regimen (daily dosing as opposed to weekly dosing with mefloquine) and gastrointestinal adverse effects [21]. Tunisia is another country that has eliminated malaria since 1979. It sends its soldiers for overseas missions in sub-Saharan Africa. In a hospital-based study that assessed the outcome of soldiers diagnosed with malaria upon return from 1993 to 2011, 37 cases were identified. Chemoprophylaxis was taken by only 21 individuals (21/37, 41 %) and non-adherence was found as the main reason for imported malaria [22]. The compliance rates reported in this study were better than those reported in studies elsewhere.

To combat imported malaria it is important to learn the strategies employed by other countries. China has suffered an increase in imported malaria from 2010 to 2014 mainly due to expatriates working abroad. Zhou et al. [23] describes that chemoprophylaxis alone is inadequate to stem the increase, and better awareness and education regarding the problem is urgently needed for people working overseas [23]. China also shares land borders with many countries and lapses in border control make it difficult to define cases of imported malaria from indigenously transmitted cases [23, 24]. Sri Lanka being an island does not have such a problem in case definition. Greece had been declared malaria free in 1974 until an outbreak of vivax malaria occurred in Evrotas in Southern Greece (a region with a large migrant population from malaria endemic countries) in 2011–12 [25]. A strategy of mass drug administration (chloroquine plus primaquine) for migrants was coupled with active case detection, community education and vector control measures. No cases of malaria were reported in Evrotas in 2013–14 [25]. Spain and Iran are two other countries seeing a surge in imported malaria with immigrant influx from North Africa and Afghanistan respectively [26, 27]. Education of migrants or visitors at border points, allowing them access to voluntary screening and preventive or curative health services have worked in Saudi Arabia, Thailand and Namibia [28–30]. Though it incurs a cost to the host country, on the -erm it can be an investment to avoid the costs of a malaria outbreak. Despite the large number of visitors received in Saudi Arabia on annual Haj pilgrimage from around the world, the country has successfully worked towards malaria elimination [28]. Regional collaboration between neighbouring countries is another effective measure to combat cross border malaria. The best example for this would be the Lubombo Spatial Development Initiative, which supported insecticide use in Mozambique and Swaziland, which in return reduced the malaria incidence in neighbouring South African districts by more than 75 % [31]. A similar network between countries providing peace-keeping forces and their hosts for sharing of knowledge and healthcare costs of treatment, chemoprophylaxis, surveillance and other resources would be beneficial to all concerned.

### Limitations

This study was based on self-reported compliance rates and adverse effect profiles. Unfortunately there were no medical records to verify these claims. While some significant associations for poor compliance were noted, the exact cause for these observations could not be established. Such an analysis would require an in depth qualitative study which was not the objective. Security forces are, by nature, hierarchical institutions and the fear of punishment in case of admitting non-compliance might have biased the reported compliance rates. The sample was dominated by respondents from the Army, which also happens to have a good health education and surveillance programme in place. The observed compliance rates might have been different if the sample had an equal number of participants from all security forces.

## Conclusions

In this study of Sri Lankan security forces personnel returning home from serving in overseas malaria endemic territories, the rate of malaria infections was found to be very low. The self-reported compliance with chemoprophylaxis was also satisfactory. A coordinated effort between the individual security forces and the National Anti Malaria Campaign ensured that security forces personnel were educated prior to departure, provided with appropriate chemoprophylaxis and screened upon re-entry to the country helping to keep the number of imported malaria cases to a minimum in this high-risk group. Such a coordinated effort is highly recommended for other countries contributing peacekeeping forces to malaria endemic countries to avoid cases of imported malaria.

## Abbreviation

AMC: Anti Malaria Campaign
